# Temperature-dependent complex dielectric permittivity: a simple measurement strategy for liquid-phase samples

**DOI:** 10.1038/s41598-023-45049-8

**Published:** 2023-10-24

**Authors:** Montgomery Baker-Fales, José D. Gutiérrez-Cano, José M. Catalá-Civera, Dionisios G. Vlachos

**Affiliations:** 1 Department of Chemical and Biomolecular Engineering, University of Delaware, 150 Academy Street, Newark, DE 19716 USA; 2 Institute of Information and Communication Technologies (ITACA), Universitat Politècnica de València, 46022 Valencia, Spain; 3 Catalysis Center for Energy Innovation, RAPID Manufacturing Institute, and Delaware Energy Institute (DEI), University of Delaware, 221 Academy St., Newark, DE 19716 USA

**Keywords:** Chemical engineering, Characterization and analytical techniques

## Abstract

Microwaves (MWs) are an emerging technology for intensified and electrified chemical manufacturing. MW heating is intimately linked to a material’s dielectric permittivity. These properties are highly dependent on temperature and pressure, but such datasets are not readily available due to the limited accessibility of the current methodologies to process-oriented laboratories. We introduce a simple, benchtop approach for producing these datasets near the 2.45 GHz industrial, medical, and scientific (ISM) frequency for liquid samples. By building upon a previously-demonstrated bireentrant microwave measurement cavity, we introduce larger pressure- and temperature-capable vials to deduce temperature-dependent permittivity quickly and accurately for vapor pressures up to 7 bar. Our methodology is validated using literature data, demonstrating broad applicability for materials with dielectric constant ε' ranging from 1 to 100. We provide new permittivity data for water, organic solvents, and hydrochloric acid solutions. Finally, we provide simple fits to our data for easy use.

## Introduction

Microwaves (MWs) are an emerging technology for chemical manufacturing. Their rapid and selective heating using renewable energy sources, like solar and wind power, can decarbonize chemical processes while enhancing performance^1–8^. MWs operate through dipole polarization and ionic conduction, depositing energy volumetrically and enabling selective heating based on the dielectric properties of materials^9^. This unique heating has been applied to various chemical processes, including organic synthesis^3,10–13^, catalytic reactors^14,15^, microfluidic processes^15–17^, nanoparticle synthesis^18–20^, polymerization reactions^21,22^, and reactive distillation^23,24^. In many such studies, the focus has been on MW-mediated process improvement or reactor design, especially as MWs expand beyond the laboratory scale^25–27^. Particularly, MW-assisted continuous flow is a promising process intensification and electrification technology for sustainable chemical manufacturing^28^.

The phenomena of MW heating are intimately linked to a material’s dielectric permittivity. The complex permittivity (ε*) of a material comprises a real part (ε′) related to polarization and stored energy, and an imaginary part (εʺ) linked to energy loss. These aspects depend on the material's structure and molecular interactions, influencing its response to external fields. As such, multiphysics simulations, such as COMSOL, have emerged for implementing MW-heated flow reactors. These simulations can optimize reactor geometry^29–31^, comprehend the impact of dielectric properties^27,31,32^, and select optimal operating experimental conditions^27^. This joint approach can drive remarkably high heating efficiency (> 95%)^27,31^ and optimal reaction performance^27^. The profound influence of temperature and pressure on the dielectric permittivity of various materials highlights the immediate significance of quantifying properties across temperatures^33–35^. Regrettably, detailed datasets are not available.

The measurement of liquid-phase dielectric permittivity at elevated temperatures poses technical challenges as elevated pressures must be accommodated to prevent boiling. Previous approaches involved constructing specialized steel pressure vessels for measuring the dielectric constant (ε′), but many of these systems could not capture the complex permittivity (they neglect the dielectric loss factor εʺ)^36–38^. Dimitrakis and coworkers demonstrated a great traceable measurement system for complex dielectric permittivity in liquids at high temperatures and pressures, utilizing a dedicated vector network analyzer (VNA) and a custom high-pressure adapter for the coaxial line^35^. Such an approach yields measurements over a large frequency range (30 kHz to 6 GHz), but the equipment is uncommon in process-oriented laboratories. The unavailability of datasets for MW heating simulations arguably stems from a disparity between the necessary equipment and the familiarity with or access to such equipment by MW practitioners. Gutierrez-Cano^39^ and coworkers report a benchtop standalone dielectric measurement kit that partly addresses this gap enabling easy room temperature, open-to-atmosphere measurements at MW-relevant frequencies ranging from 1.5 to 2.6 GHz^39^.

In this study, we present an extension to the benchtop dielectric measurement kit described by Gutierrez-Cano^39^ that enables pressurization and temperature resolution to rapidly acquire the temperature-dependent complex dielectric permittivity near the 2.45 GHz ISM frequency relevant to MW heating.

## Methods

The experimental setup employed in this study is a modified system^39^, an autonomous system capable of acquiring dielectric properties of materials near the 2.45 GHz ISM band. This design allows convenient measurement of materials inside tubes, particularly suitable for liquids, semisolids, powders, and granular materials. The MW structure is based on a single-post re-entrant cavity^40^, CNC-machined from aluminum (± 5 µm precision), and featuring an open port on the top cover to accommodate the tubes along the central axis of the cell. The measurement procedure relies on the precise measurement of the MW cavity’s reflection or impedance to determine the resonant frequency and quality factor and the subsequent use of the resonance parameters to calculate the dielectric properties of the material from a rigorous numerical method^39^. Permittivity characterization with MW cavities is typically restricted to low-loss materials^41^. However, high-coupling networks enable measurements of both low-loss and high-loss materials using the same experimental setup. To streamline and simplify the measurement process, the device includes a tailor-made MW reflectometer, which assesses the MW cavity's reflection coefficient, eliminating the need for a full-featured VNA.

The developed electromagnetic (EM) numerical method of the MW cavity to calculate the dielectric properties of the materials made use of circuit analysis and mode-matching techniques^42^, as described in detail in previous work^39^. This technique involves decomposing the complex MW cavity into simpler canonical circuits, which are analyzed and computed independently and then connected to assemble the complete structure. The relationship between the complex resonance frequency (resonant frequency and quality factor) and complex permittivity (dielectric constant and loss factor) is solved by applying the resonance condition to the numerical response of the MW cavity. Unlike other dielectric properties measurement methods, such as the cavity perturbation methods (CPM), the approach described in reference^39^ does not require calibration with reference materials. We refer readers to our previous demonstration of such a system for a full description^39^ and that we build upon here.

**Figure 1:**
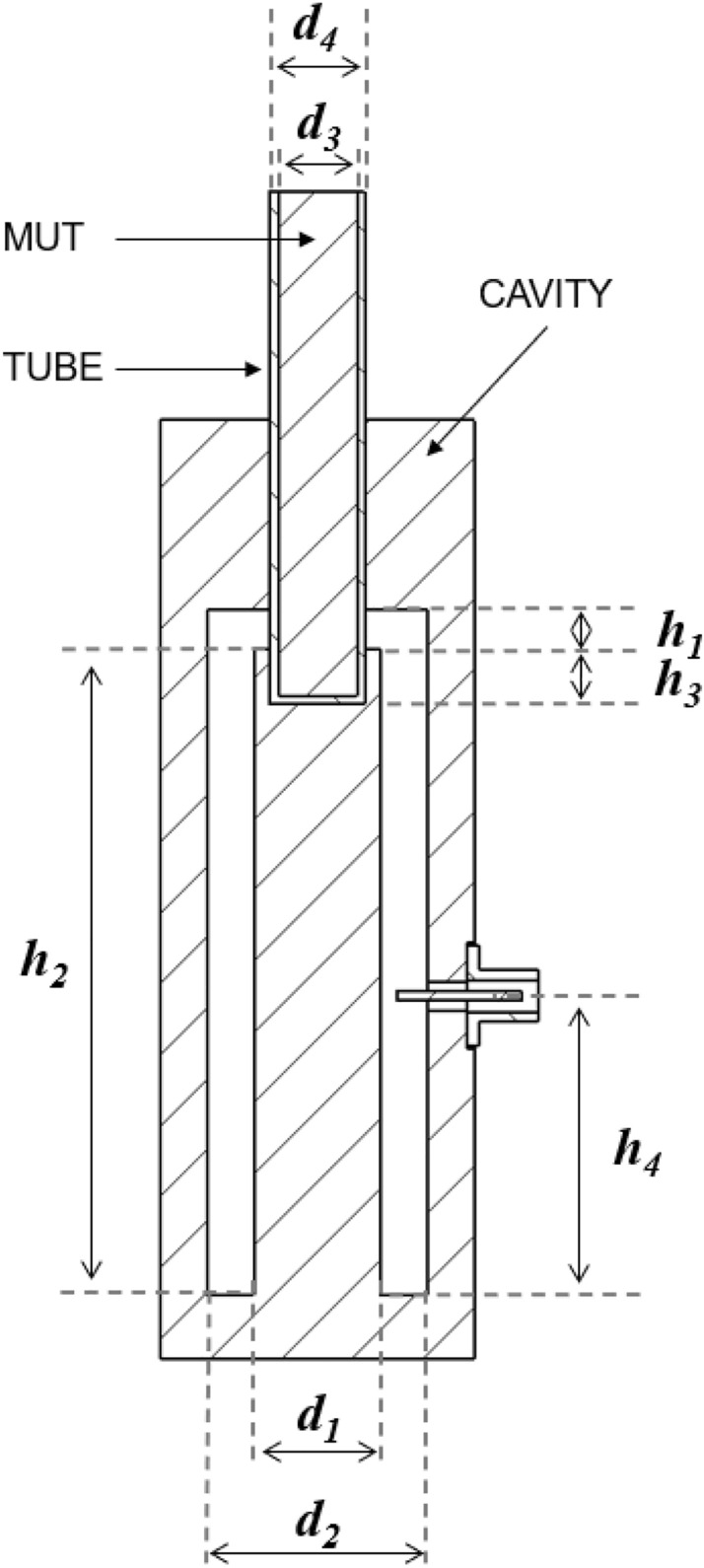
Cross-section of the designed bireentrant MW cavity, tube, and material under test (“MUT”) with dimensions h_1_ = 5 mm, h_2_ = 82 mm, h_3_ = 6 mm, h_4_ = 41 mm, d_1_ = 16.1 mm, d_2_ = 28 mm, d_3_ = 10 mm, and d_4_ = 12 mm.

In the previous setup, commercial Pyrex tubes were utilized to house the materials. However, the permittivity of Pyrex is highly temperature-dependent^43^. As the temperature rises, the resonance measurement becomes influenced by both the changes in material permittivity and the variations in the tube's permittivity. This interplay complicates the development of an accurate EM model and its practical implementation. On the other hand, while the small size of the Pyrex tubes facilitates the use of minimal sample quantities, it limits the incorporation of external elements for high-pressure containment or atmospheric control. To overcome these limitations, an upgraded MW cavity has been developed to accommodate more convenient larger tubes, with the dimensions of the tubes and cavity described in Fig. 1. These tubes are crafted from materials like quartz and alumina, which exhibit more consistent permittivity across different temperatures^44,45^.

**Figure 2:**
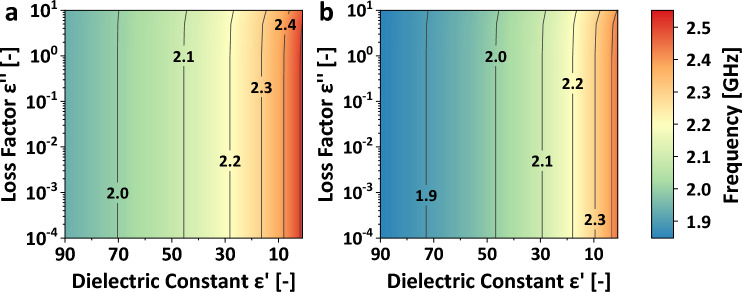
Frequency shift as a function of permittivity for the designed bireentrant cavity. (**a**) Dielectric materials enclosed in quartz tubes and (**b**) dielectric materials inside alumina tubes. Contours are placed at 0.1 GHz frequency increments.

An EM numerical model was developed in MATLAB and employed to ascertain the optimal dimensions of the bireentrant resonator, aiming to achieve a sensitivity level comparable to that of the Pyrex tube version, attaining a single resonant mode encompassing the operational range of the MW reflectometer, spanning from 1.5 to 2.6 GHz. Figure 1 depicts a cross-sectional view of the resonant geometry and the dimensions. The relationship between the permittivity and the resonant frequency of the resulting bireentrant MW structure is shown in Fig. 2. Figure 2a illustrates the frequency shift of the cavity loaded with quartz tubes, and Fig. 2b corresponds to the frequency variation using alumina tubes. The resonance frequencies of the cavity using alumina tubes for the same dielectric material are lower than those using quartz tubes, attributed to the higher dielectric constant of alumina. Nevertheless, in both cases, a maximum frequency shift of approximately 600 MHz is achieved for materials with a dielectric constant ranging from 1 to 100, like the frequency shift obtained with the Pyrex tubes in the original bireentrant setup^39^.

**Figure 3:**
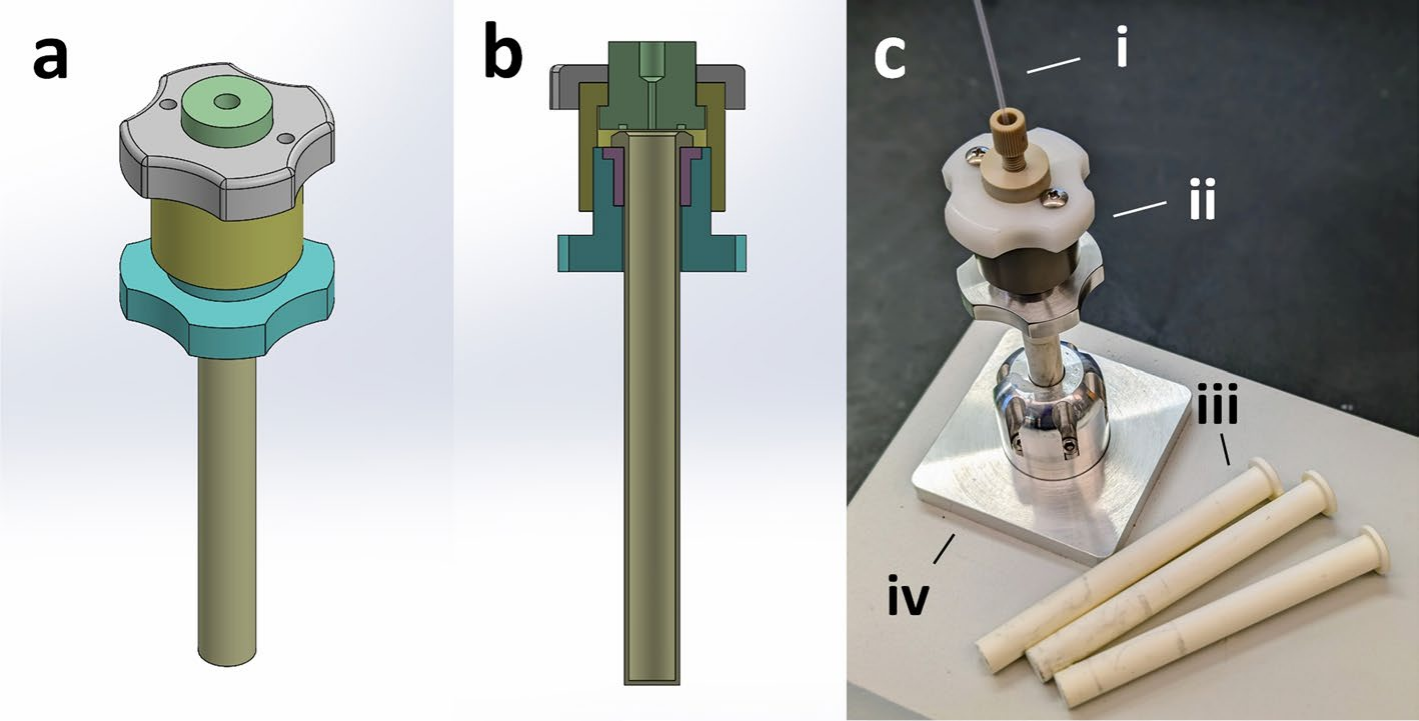
Depiction of the PTD vessel. The external (**a**) and internal cross section (**b**) views of the vessel are shown as designed for machining, illustrating the simplicity of the approach. A photograph of the vessel (**c**) with the temperature-sensing optical fibers (i), screw-topped sealing fixture (ii), alumina vials (iii), and the cavity access hole (iv).

The described MW setup allows for flexible sample handling. A vessel was then developed to facilitate high-pressure containment of liquid samples at elevated temperatures while still allowing for accurate measurement of dielectric permittivity. To maintain permittivity uncertainties within a 2% margin for the dielectric constant and a 5% margin for the loss factor, the uncertainty associated with the internal diameter of the tube, with respect to the nominal value used in the model, was determined to be ± 20 µm^39^. The pressure–temperature-dielectric (PTD) vessel is illustrated in Fig. 3 and fulfills the requirements as follows: Alumina vials (Fig. 3c(iii)) are designed to fit the access hole of the bireentrant cavity (iv) and are equipped with a flanged top for sealing via an o-ring fixture. A threaded assembly (ii) ensures pressure containment within the vessel. Additionally, a temperature-sensing optical fiber (i) with a silica cladding is inserted and sealed within the vial using soft ETFE ferrules, as described in prior work^31,46^. Specific dimensions of the alumina vial are offered in Fig. S1. The PTD vessel is heated to a predetermined temperature using an external heating block, after which it is transferred to the cavity. As the vessel cools, permittivity-temperature data points are collected. Throughout this process, the pressure changes to reflect the vapor pressure of the solvent at each temperature. We do not expect this to affect the measurements for sub-critical liquids as demonstrated by Dimitrakis and coworkers^35^ who showed no difference in the permittivity for alcohols at pressures ranging from 1 to 60 bar.

## Results and discussion

### Validation of permittivity measurements

Although the MW setup can operate with both quartz or alumina tubes which result in nearly identical measurements (Fig. S2), quartz tubes are limited to near atmospheric pressures. This study focuses on alumina vials since they have superior pressure resistance and can reliably be used up to 7 bar, providing access to wider temperature ranges for liquid permittivity measurements.

**Figure 4:**
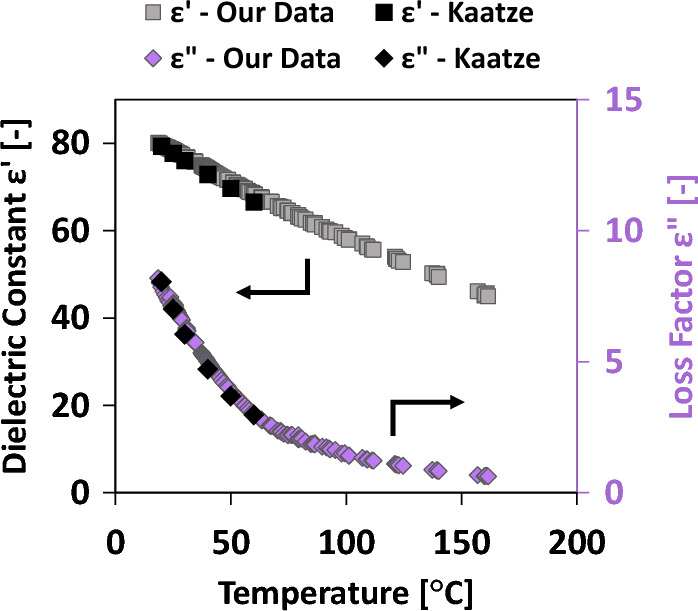
Temperature-dependent dielectric permittivity of water. Comparison of literature values with measurements made using alumina vials. Square symbols represent ε′ values (left axis) while diamond symbols represent εʺ values (right axis).

To validate the temperature measurement scheme, the dielectric permittivity of ultrapure water was measured from 20 to 160 °C. While some references in the literature report water permittivity up to 100 °C^33,34^, the frequency at which they provide the permittivity values is significantly different. This discrepancy hinders the proper comparison of permittivity values due to the strong dependency of permittivity with frequency for polar materials^47^. Then the measured permittivity was compared to the values given by the Debye-type relaxation spectral function provided by Kaatze^48^, allowing the calculation of the permittivity of water as a function of frequency and temperature up to 60 °C. Figure 4 compares our measurements taken at 1.85 GHz to the permittivity values obtained from Kaatze's Debye model at the same frequency. Our experimental results align remarkably well with those values throughout the entire temperature range of the reference^48^. This agreement in data demonstrates the successful efficacy of our temperature measurement scheme in accurately capturing the temperature-dependent dielectric permittivity.

**Figure 5:**
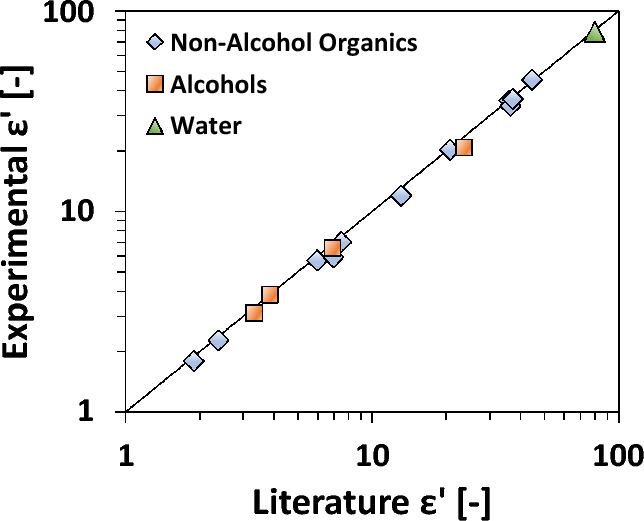
Parity of measured and literature ε′ values for various solvents at room temperature utilizing the alumina PTD vessel. Including data from references^49–59^.

To demonstrate the efficacy of alumina vials for measuring various solvents across a wide range of ε′ values, we conducted room temperature dielectric constant measurements as depicted in Fig. 5. We compare our experimental results to literature values obtained from different sources (refer to Table S1 for details). Notably, certain solvents exhibit a significant ε' variation depending on the measurement frequency according to literature data (namely alcohols). After accounting for this, the agreement between our experimental measurements and the reported literature values is excellent (an average of 5.8% relative error) across the entire ε' range of approximately 1 to 100. Unfortunately, literature data for solvents at elevated temperatures and the same frequency as our measurements are lacking, so additional comparisons cannot be made.

In comparison to the most contemporary approach^35^, our experimental setup does not require a full-featured VNA, an HPLC pump, a dedicated temperature controller, or a customized high-pressure adapter for the coaxial line. Rather, temperature is continuously monitored internally in the alumina vial which is placed on a hot plate for heating and then removed and placed into the measurement cell at the desired time. This process can be repeated quickly for various liquid samples, and the experimental data presented in this work was collected in a single day. However, our approach can only reach moderate pressures (~ 7 bar) with the current design which can limit the accessible temperature range. Furthermore, our method does not allow for traceable measurements which may be important given the sensitivity to frequency exhibited by certain compounds. The low experimental variance in our measurements also represents an improvement, possibly owing to our seal placement scheme which avoids the introduction of geometric changes to the measurement cell upon pressurization^35^.

### Dielectric permittivity of non-alcohol organic solvents

**Figure 6:**
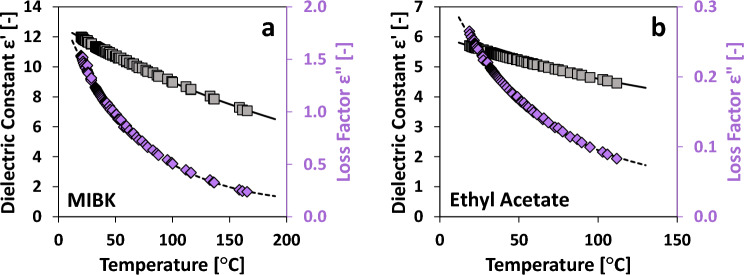
Sample permittivity profiles for MIBK (**a**) and ethyl acetate (**b**). Grey squares represent ε′ (left axis) while purple diamonds represent εʺ (right axis). Solid and dashed lines represent the exponential fits for ε′ and εʺ data, respectively.

We tested a variety of organic solvents to investigate their temperature-dependent permittivity behavior and establish a small repository of such data. Most solvents generally exhibit a consistent, monotonic decrease in both ε' and ε" as temperature increases. Figure 6a and Fig. 6b showcase this trend with representative examples of MIBK and ethyl acetate permittivity. For such cases, a straightforward three-parameter exponential fit, as presented in Eq. (1), provides an accurate, empirical model of permittivity across the entire range of measured temperatures.

1 $$\varepsilon ={A}_{1}\cdot \mathrm{exp}\left(-{A}_{2}\cdot T\right)+{A}_{3}$$

Here, A_1_, A_2_, and A_3_ are the regressed parameters, T is the measured temperature [°C], and ε is either the real or imaginary component of the dielectric permittivity. The temperature-dependent permittivity data for each non-alcohol organic solvent is listed in Figs. S3 and S4, while the regressed parameters A_1_, A_2_, and A_3_ from Eq. (1) are available for each solvent in Table 1. The standard error of the fitted Eq. (1) for each solvent is less than 3% in all cases. The suitable temperature range for each solvent was chosen to limit the vapor pressure to 7 bar for safe operation.

**Table 1:** Regressed parameters for non-alcohol organic solvents fitted to Eq. (1).

Solvent	Dielectric constant ε′ [–]				Loss factor εʺ [–]				T_MAX_
	A_1_	A_2_	A_3_	Error	A_1_	A_2_	A_3_	Error	
MIBK	12.801	− 0.003555	0.000	0.5%	1.908	− 0.015495	0.095	1.9%	165.4
Tetrahydrofuran	7.089	− 0.003233	0.326	0.3%	0.219	− 0.012762	0.025	1.0%	135.4
γ-Valeractone	35.766	− 0.001980	0.000	1.9%	12.924	− 0.017054	1.041	2.6%	185.3
DMSO	48.082	− 0.002262	0.000	0.9%	14.008	− 0.019674	1.223	1.4%	179.0
Dimethylformamide	29.870	− 0.005903	9.803	0.3%	5.954	− 0.017535	0.481	1.7%	186.6
Ethyl acetate	3.537	− 0.005128	2.484	0.1%	0.299	− 0.017324	0.042	0.9%	111.8
Acetonitrile	33.160	− 0.004736	5.434	0.2%	1.737	− 0.014391	0.207	0.6%	133.8
2 m-THF	4.642	− 0.004662	1.673	0.2%	0.289	− 0.014894	0.024	0.8%	145.2
Acetone	22.997	− 0.004106	− 0.936	0.3%	1.036	− 0.013575	0.059	0.8%	115.4

The standard error relative to the mean is reported for each parameter set along with the maximum temperature measured for each solvent.

### Dielectric permittivity of alcohols

**Figure 7:**
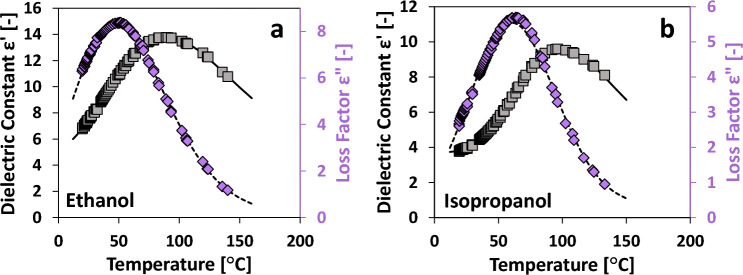
Sample permittivity profiles for ethanol (**a**) and isopropanol (**b**). Grey squares represent ε′ (left axis) while purple diamonds represent εʺ (right axis). Solid and dashed lines represent the lognormal fits for ε′ and εʺ data, respectively.

The permittivity of alcohols displays a nonmonotonic dependence on temperature^35,60^. As temperature increases, so do ε′ and εʺ until the trend is reversed at some point and ε′ and εʺ decrease with further increments of temperature. This effect is highlighted in Fig. 7a and Fig. 7b where ethanol and isopropanol permittivity are displayed. In these cases, a modified lognormal fit is used with a total of 6 regressed parameters as shown in Eq. (2)

2 $$\varepsilon =\frac{{B}_{2}}{{B}_{1}+T}\cdot \frac{{B}_{3}}{\sigma \sqrt{2\pi }}\cdot \mathrm{exp}\left(-\frac{{\left(\mathrm{ln}\left(\frac{{B}_{1}+T}{{B}_{2}}\right)-\mu \right)}^{2}}{2{\sigma }^{2}}\right)+{B}_{4}$$

where B_1_, B_2_, B_3_, B_4_, σ, and µ are the regressed parameters. In this case, σ and µ are the traditional descriptors of a lognormal fit while B_1_, B_2_, B_3_, and B_4_ allow x-axis and y-axis stretching and offset. The temperature-dependent permittivity data for each alcohol are listed in Fig. S5, while the regressed parameters B_1_, B_2_, B_3_, B_4_, σ, and µ from Eq. (2) are available for each solvent in Table 2. The standard error of the fitted Eq. (2) for each solvent is less than 3.5% in all cases.

**Table 2:** Regressed parameters for alcohols fitted to Eq. (2).

Solvent	B_1_	B_2_	B_3_	B_4_	σ	µ	Error	T_MAX_
Dielectric constant ε′ [–]								
Ethanol	112.82	169.75	6.846	5.167	0.23079	0.25746	0.6%	140.0
Methanol	17.572	195.77	36.847	0.8127	1.01634	0.00305	0.2%	111.5
Isopropanol	89.533	137.39	4.275	3.681	0.20388	0.35941	0.8%	133.0
2-Pentanol	27.006	108.26	3.239	3.102	0.33902	0.30006	1.1%	172.1
Loss factor εʺ [–]								
Ethanol	177.08	193.78	4.516	0.106	0.17261	0.17374	1.0%	140.0
Methanol	315.885	754.41	2645.101	0.0000	0.53719	-2.59687	0.8%	111.5
Isopropanol	561.926	324.10	1.352	0.317	0.05364	0.65545	2.6%	133.0
2-Pentanol	313.805	288.81	0.752	0.109	0.08754	0.27231	3.4%	172.1

The standard error relative to the mean is reported for each parameter set along with the maximum temperature measured for each solvent.

### Dielectric permittivity of aqueous hydrochloric acid solutions

**Figure 8:**
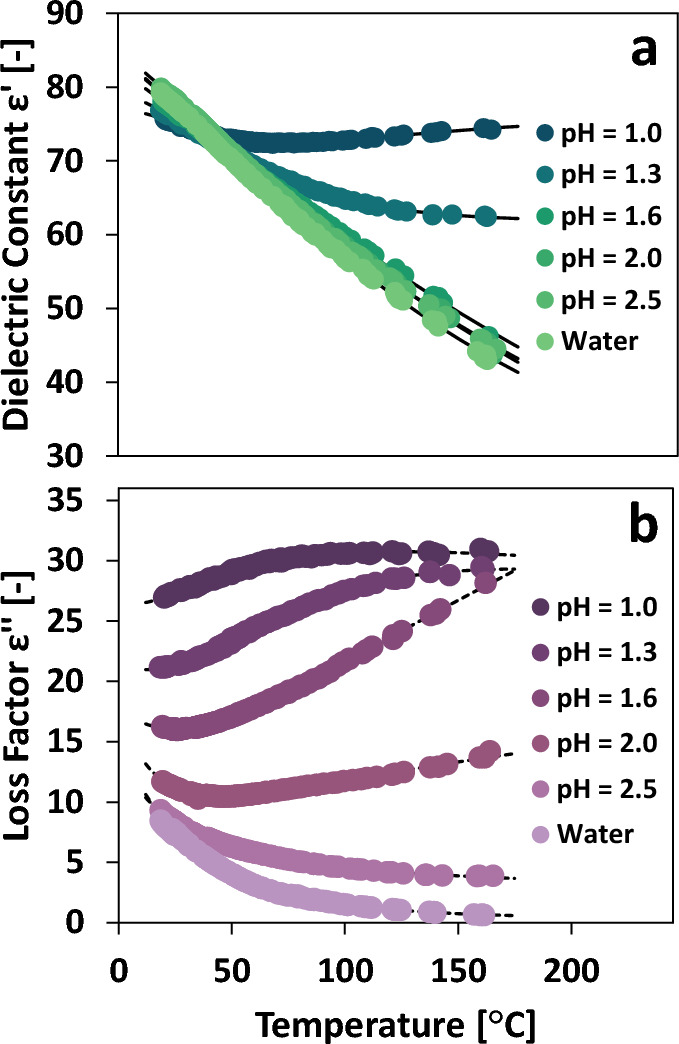
Temperature-dependent dielectric permittivity of hydrochloric acid solutions including dielectric constant (**a**) and loss factor (**b**).

Next, we measured permittivity of water and hydrochloric acid solutions of various strengths. Figure 8a shows the permittivity for a continuum of HCl solutions ranging from pH 7 to 1. ε′ for pure water descends monotonically with temperature. Up to pH ≈ 1.6, HCl addition has little effect on εʺ. However, for stronger and stronger HCl solutions, smaller and smaller ε' drops are observed with increasing temperature. Finally, at pH 1, ε′ increases with increasing temperature. ε′ at room temperature decreases slightly by lowering pH. In Fig. 8b, εʺ is reported for the same solutions. HCl addition monotonically increases εʺ—even at room temperature. As HCl is added, the decrease in εʺ with temperature is less prominent until pH 2, where increases in temperature cause increases in εʺ. This behavior in which ionic concentration changes the temperature effect on permittivity has not been previously reported to our knowledge.

The permittivity data for HCl solutions is fitted using Eq. (2). The regressed parameters for each HCl solution are presented in Table 3. In each case, the standard error of the fitted Eq. (2) is less than 4%.

**Table 3:** Regressed parameters for HCl solutions fitted to Eq. (2).

Solution	B_1_	B_2_	B_3_	B_4_	σ	µ	Error	T_MAX_
Dielectric constant ε′ [–]								
Water	328.07	440.42	50.052	20.425	0.4137	− 0.35783	0.3%	161.5
pH 2.5	100.153	251.06	133.19	0.4520	1.1311	0.00290	0.2%	165.4
pH 2.0	257.88	527.20	64.293	4.604	0.6550	− 0.63567	0.3%	164.0
pH 1.6	254.28	437.25	103.07	− 4.812	0.7801	− 0.32871	0.2%	162.1
pH 1.3	170.802	93.591	22.15	61.793	0.2754	0.61419	0.2%	161.3
pH 1.0	3.389	100.943	− 7.424	76.750	0.7132	0.21673	0.1%	163.3
Loss factor εʺ [–]								
Water	76.014	84.415	61.390	0.24264	0.8689	− 1.1598	3.0%	161.5
pH 2.5	37.13	10,586.3	2630.04	2.4934	13.5644	− 58.4632	1.1%	165.4
pH 2.0	5.0850	253.13	− 37.596	23.493	1.6017	0.9550	0.7%	164.0
pH 1.6	49.807	765.6	− 177.51	93.607	1.8130	0.9576	0.3%	162.1
pH 1.3	− 0.12798	246.07	15.783	20.938	0.7880	0.2661	0.4%	161.3
pH 1.0	− 0.12801	218.71	8.8847	26.337	0.9260	0.2853	0.3%	163.3

The standard error relative to the mean is reported for each parameter set along with the maximum temperature measured for each solvent.

## Conclusions

We demonstrate a simple benchtop approach for evaluating temperature-dependent dielectric permittivity near the 2.45 GHZ ISM frequency, constituting a substantial simplification of existing equipment and ease of use. By building upon the existing benchtop standalone dielectric measurement kit with larger, easier-to-use vials, a simple pressurization scheme was implemented. Improvements in vial materials were also made by tuning the electromagnetic model for quartz and alumina tubes, which exhibit less sensitivity of dielectric permittivity to temperature. Ultimately, liquids can be heated via a hotplate to temperatures significantly above their boiling point and up to 7 bar, and the pressurized and heated vial can be transferred to the measurement cavity for permittivity evaluation. The methodology was validated using the known temperature-dependent permittivity of water and the room temperature permittivity of various solvents. Excellent agreement with the literature data was demonstrated.

We then report the permittivity of water, alcohols, organic solvents, and hydrochloric acid solutions across temperatures up to 7 bar of vapor pressure. While water and non-alcohol organic solvents display monotonic decreases in ε′ and εʺ with increasing temperatures, more surprising behavior emerges for other compounds. Alcohols seemingly share a separate behavior in which ε′ and εʺ increase to a maximum and then decrease with increasing temperature. Hydrochloric acid solutions also exhibit non-monotonic behavior which changes across pH values. Based on the permittivity behavior for the various categories of liquids, exponential and lognormal fits were provided.

### Supplementary Information

Supplementary Information 1.

Supplementary Information 2.

## Data Availability

The datasets used and/or analyzed during the current study are available from the corresponding author upon reasonable request.
