# Supplementary material for: Temperature-dependent complex dielectric permittivity: a simple measurement strategy for liquid-phase samples
Source: Sci Rep. 2023 Oct 24;13:18171. doi: 10.1038/s41598-023-45049-8 (PMC10597996; doi:10.1038/s41598-023-45049-8)
Supplement: Supplementary file 2 — Supplementary Information 2. [file 41598_2023_45049_MOESM2_ESM.docx]

Supplementary Information

Temperature-Dependent Complex Dielectric Permittivity:
A Simple Measurement Strategy for Liquid-Phase Samples

Montgomery Baker-Fales^1†^, José D. Gutiérrez-Cano^2^, José M. Catalá-Civera^2^, and Dionisios G. Vlachos^1,3,*^

^1^Department of Chemical and Biomolecular Engineering, University of Delaware, 150 Academy Street, Newark, Delaware 19716, United States

^2^Institute of Information and Communication Technologies (ITACA), Universitat Politècnica de València, 46022 Valencia, Spain

^3^Catalysis Center for Energy Innovation, RAPID Manufacturing Institute, and Delaware Energy Institute (DEI), 221 Academy St., University of Delaware, Newark, Delaware 19716, United States

*Corresponding author: [vlachos@udel.edu](mailto:vlachos@udel.edu)

# Alumina Vial Schematic


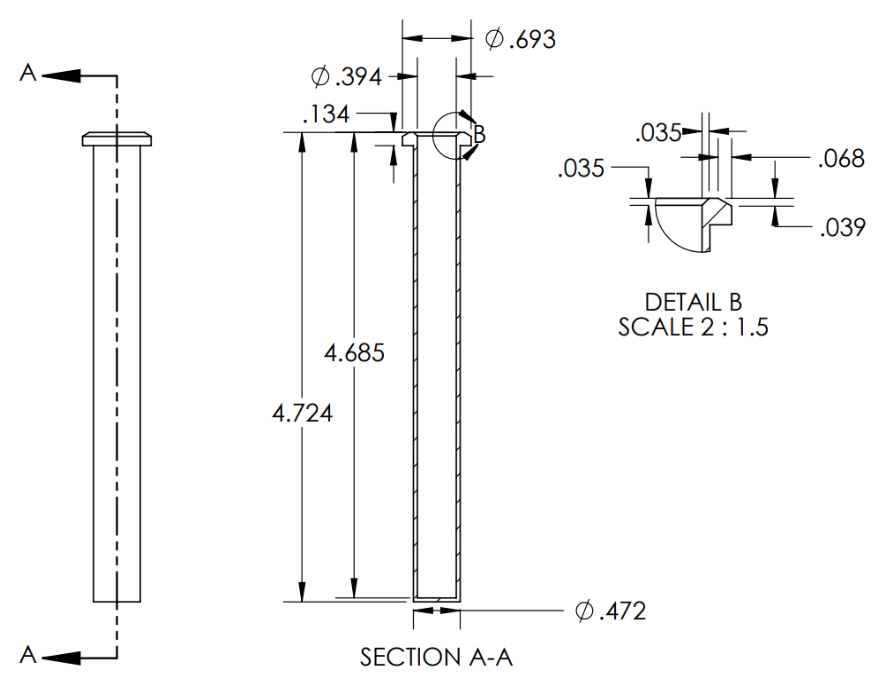


Figure S1. Schematic of alumina vials for PTD vessel with dimensions in inches.

The alumina vials used in this study were composed of 99.7% purity Al_2_O_3_ by Precision Ceramics USA to the dimensions shown in Figure S1.

# Alumina and Quartz Vial Performance Comparison

In Figure S2, we compare the temperature-dependent dielectric permittivity of ultrapure water using quartz and alumina vials. Alumina vials, being more pressure-resistant, have been reliably utilized up to 7 bar, granting access to wider temperature ranges. As depicted in Figure S2, both ε' and ε" exhibit nearly identical measurements in quartz and alumina vials. Some variations in ε" values at lower temperatures are attributed to the slight variation in measurement frequency observed with alumina vials (1.85 GHz) and the frequency sensitivity of ε". Hence, alumina vials effectively expand the range of achievable temperatures for liquid permittivity measurements.

Figure S2. Temperature-dependent dielectric permittivity of water. Comparison of measurements made using quartz and alumina vials.

# Experimental Permittivity Measurement Comparison to Literature

In Table S1, the experimentally measured room temperature dielectric constants are presented for a small library of solvents, where measurements were made using alumina vials. Literature values found are also presented for comparison.

Table S1. Room temperature dielectric constant measurements and comparison to literature. For solvents with a superscript^a^, a strong E’ dependence on measurement frequency was noted, and a corresponding literature source at that same frequency is reported. Literature sources are also noted as superscripts.

| **Compound** | **This Work** | | **Literature Values** |
| --- | --- | --- | --- |
|  | **E' [-]** | **f [MHz]** | **E' [-]** |
| **Hexane^1^** | 1.79 | 2398.3 | 1.89 |
| **Toluene^2^** | 2.27 | 2389.0 | 2.38 |
| **Ethyl Acetate^3^** | 5.68 | 2323.3 | 5.99 |
| **2-mTHF^4^** | 5.90 | 2319.4 | 6.97 |
| **THF^4^** | 7.03 | 2300.7 | 7.50 |
| **MIBK^5^** | 11.99 | 2226.9 | 13.11 |
| **Acetone^6^** | 20.30 | 2135.7 | 20.70 |
| **Acetonitrile^2^** | 35.77 | 2021.5 | 35.94 |
| **GVL^7^** | 33.59 | 2037.9 | 36.47 |
| **Dimethylformamide^8^** | 36.36 | 2016.4 | 37.20 |
| **DMSO^9,a^** | 45.33 | 1975.1 | 44.55 |
| **2-Pentanol^10,a^** | 3.12 | 2374.8 | 3.32 |
| **Isopropanol^9,a^** | 3.86 | 2360.0 | 3.84 |
| **Ethanol^9,a^** | 6.58 | 2303.1 | 6.90 |
| **Methanol^9,a^** | 20.89 | 2103.7 | 23.64 |
| **Water^11^** | 78.81 | 1855.5 | 80.10 |

# Dielectric Permittivity of Non-Alcohol Organic Solvents

Figure S3. Dielectric permittivity of non-alcohol organic solvents – part 1. Plots a, b, c, d, e, and f represent properties of MIBK, tetrahydrofuran, γ-valeractone, dimethyl sulfoxide, dimethyl formamide, and ethyl acetate, respectively. Grey squares and purple diamonds and represent ε' and ε" data, respectively.

Figure S4. Dielectric permittivity of non-alcohol organic solvents – part 2. Plots g, h, and i represent properties of acetonitrile, 2-methyl-tetrahydrofuran, and acetone, respectively. Grey squares and purple diamonds and represent ε' and ε" data, respectively.

# Dielectric Permittivity of Alcohols

Figure S5. Dielectric permittivity of alcohols. Plots a, b, c, and d represent properties of ethanol, methanol, and isopropanol, and 2-pentanol, respectively. Grey squares and purple diamonds and represent ε' and ε" data, respectively.

# References

1. Mopsik, F. I. Dielectric constant of n-hexane as a function of temperature, pressure, and density. *J Res Natl Bur Stand A Phys Chem* **71A**, (1967).

2. Ritzoulls, G., Papadopoulos, N. & Jannakoudakls, D. Densities Viscosities, and Dielectric Constants of Acetonitrile + Toluene at 15, 25, and 35 °C. *J Chem Eng Data* **31**, (1986).

3. Sastry, N. V. & Patel, M. C. Densities, excess molar volumes, viscosities, speeds of sound, excess isentropic compressibilities, and relative permittivities for alkyl (methyl, ethyl, butyl, and isoamyl) acetates + glycols at different temperatures. in *Journal of Chemical and Engineering Data* vol. 48 (2003).

4. Aycock, D. F. Solvent applications of 2-methyltetrahydrofuran in organometallic and biphasic reactions. *Org Process Res Dev* **11**, (2007).

5. Tsierkezos, N. G., Kelarakis, A. E. & Molinou, I. E. Densities, viscosities, refractive indices, and surface tensions of 4-methyl-2-pentanone+ethyl benzoate mixtures at (283.15, 293.15, and 303.15) K. *J Chem Eng Data* **45**, (2000).

6. De Jesús-González, N. E., Pérez De La Luz, A., López-Lemus, J. & Alejandre, J. Effect of the Dielectric Constant on the Solubility of Acetone in Water. *J Chem Eng Data* **63**, (2018).

7. Shen, X. R., Xia, D. Z., Xiang, Y. X. & Gao, J. G. γ-valerolactone (GVL) as a bio-based green solvent and ligand for iron-mediated AGET ATRP. *E-Polymers* **19**, (2019).

8. Hunger, J. *et al.* Relative permittivity of dimethylsulfoxide and N, N -dimethylformamide at temperatures from (278 to 328) K and pressures from (0.1 to 5) MPa. *J Chem Eng Data* **55**, (2010).

9. Gregory, A. P. & Clarke, R. N. Tables of the Complex Permittivity of Dielectric Reference Liquids at Frequencies up to 5 GHz; NPL Report MAT 23. *Innovation* (2009).

10. Shinomiya, T. Dielectric Dispersion and Intermolecular Association for 28 Pure Liquid Alcohols. The Position Dependence of Hydroxyl Group in the Hydrocarbon Chain. *Bull Chem Soc Jpn* **62**, (1989).

11. Malmberg, C. G. & Maryott, A. A. Dielectric constant of water from 0 to 100 C. *J Res Natl Bur Stand (1934)* **56**, (1956).
